# Scattering of surface waves by a vertical truncated structured cylinder

**DOI:** 10.1098/rspa.2021.0824

**Published:** 2022-02

**Authors:** R. Porter, S. Zheng, H. Liang

**Affiliations:** ^1^ School of Mathematics, Woodland Road, University of Bristol, Bristol, BS8 1UG, UK; ^2^ School of Engineering, Computing and Mathematics, University of Plymouth, Drake Circus, Plymouth PL4 8AA, UK; ^3^ State Key Laboratory of Coastal and Offshore Engineering, Dalian University of Technology, Dalian, 116024, China; ^4^ Technology Centre for Offshore and Marine, Singapore (TCOMS), 118411, Singapore

**Keywords:** plate arrays, vertical cylinders, water waves

## Abstract

This paper describes the solution to the problem of scattering of plane incident waves on water of constant depth by a bottom mounted circular cylinder, extending partially through the depth, which has an internal structure comprised of closely spaced thin vertical barriers between which fluid is allowed to flow. The problem is solved under full depth-dependent linearized water wave theory using an effective medium equation to describe the fluid motion in cylinder and effective boundary conditions to match that flow to the fluid region outside the cylinder. The interest in this problem lies in the development of novel solution methods for fully three-dimensional water wave interaction with bathymetric plate arrays. Results computed using this theory are compared with a shallow water approximation based on the recent work of Marangos & Porter (2021 Shallow water theory for structured bathymetry. *Proc. R. Soc. A* **477**, 20210421.) and with accurate computations of an exact representation of the geometry using a discrete set of plates. Other results highlight the resonant directional lensing effects of this type of cylindrical plate array device.

## 1. Introduction

Closely spaced arrays of thin plates extending vertically from the bottom of an ideal fluid with a free surface have been used recently by a number of authors to produce unusual effects on water waves that are inaccessible using conventional changes in depth, notably the ability to negatively refract waves. Contributions include Berraquero *et al.* [1], Maurel *et al.* [2,3] and Marangos & Porter [4], all of whom assumed long wavelengths compared to the depth and subsequently developed depth-averaged models to describe the wave propagation over this plate-array structured bathymetry. Each of those models produces the same governing two-dimensional wave equation in which the conventional fluid depth is replaced by a diagonal rank two tensor but whose elements differ depending on further assumptions made about the spacing of gaps within the plate array. The two diagonal tensor entries determine the phase velocities of surface waves propagating in different directions over the bathymetry and this anisotropy leads directly to negative refractive effects. It is also an important ingredient in bathymetric cloaking of vertical cylinders [5].

The model of Marangos & Porter [4] applies to close spacing between the plates where tensor elements are expressed explicitly in terms of the distances from the surface to the top of the array of plates and to the base of the fluid. This model arises from homogenization which exploits the contrast in the lengthscales in the problem and is adopted from Porter [6] and Zheng *et al.* [7]. In these two problems, the plate arrays extend throughout the fluid depth allowing the exact depth dependence of the fluid to be factorized from the solution without depth averaging and is therefore not restricted to long wavelengths. This factorization implies the water wave equations are analogous to the equations that govern two-dimensional acoustics and polarized electromagnetics.

Whilst Porter's [6] work concentrated on refraction across planar interfaces, Zheng *et al.* [7] considered the effect on waves of plate arrays confined within a circular cylinder. The small gaps between the plates provide an environment for resonance and slow wave propagation across the cylinder giving it the ability to concentrate wave energy which can either be harnessed/dissipated via a local damping mechanism or redirected to create a water wave lensing device. Moreover, when the incident wave direction is aligned with the plates, the cylinder becomes completely transparent to waves, making the cylinder an interesting prospect for marine energy harvesting since it can be rotated to protect itself when necessary whilst possessing the potential to capture significant amounts of energy when engaged against incident waves.

In this paper, we consider the effect that truncating the vertical extent of cylinder considered by Zheng *et al.* [7] has on the scattering of incident waves. Specifically, we have assumed the cylinder extends upwards from the base of the fluid to a constant level below the surface. Although this particular configuration is not a candidate for wave energy capture, it does allow results to be compared to the shallow water model of Marangos & Porter [4]. This serves an important purpose and is one of the motivations for the present work: calibrating the conditions under which the much simpler and more widely used shallow water approximation can be used to accurately determine scattering by bathymetric plate array devices.

The structural uniformity in the depth exploited by Zheng *et al.* [7] is absent here and this introduces additional mathematical challenges not previously encountered. It is the description of this novel and bespoke solution process for the full depth-dependent model on which the emphasis of this work is placed. We regard this as an important prototype problem for developing the solution methods for more complex problems involving wave energy harvesting by multiscale devices which involve mechanical damping components. A particular motivation for considering the bottom mounted submerged truncated cylinder as opposed to cylinders intersecting the surface is to avoid problems associated with undamped resonance for sufficiently high frequencies as encountered by Zheng *et al.* [7].

Separation solutions are employed but, within the cylindrical region defined by the cylinder radius, the field equation satisfied by the velocity potential above and below the submerged level of the top of the cylinder switches from the three-dimensional Laplace equation to a reduced two-dimensional Laplace equation. Thus the expansion of the solution inside the cylindrical region is reminiscent of situations that occur when considering wave propagation in, for example, a density-stratified multi-layer fluid (e.g. [8]) in which vertical eigenfunctions are defined in a piecewise fashion and satisfy a generalized orthogonality condition and a non-standard dispersion relation. This description poses challenges. Appendix A of this paper provides a discussion of the location of roots of the dispersion relation in the complex plane which is a matter of significant practical importance. However, more technical aspects including the question as to whether the eigenfunction form a complete basis addressed, for example, in a similar setting by Lawrie [9,10] are not considered here.

The paper is laid out as follows. The problem is formulated in §2 and the full depth-dependent solution is described in §3. Section 4 applies the shallow water approximation of Marangos & Porter [4] to this particular configuration. The numerical results are presented in §5 before the work is summarized in §6.

## 2. Description of the problem

We work with a mixture of three-dimensional Cartesian and cylindrical coordinates with z=0 coinciding with the mean free surface of the fluid, which rests above a horizontal bed at z=−h. Otherwise (x,y)=(rcos⁡θ,rsin⁡θ) lie in the horizontal plane and a structured cylinder is enclosed within the region r<a, −h<z<−d, 0≤θ<2π.

**Figure 1.**
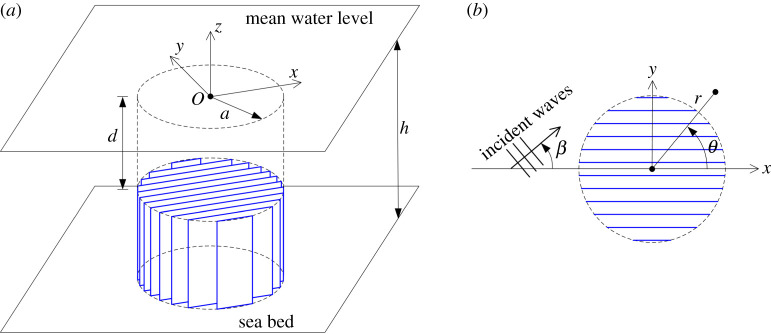
Illustration of the geometry. In (*b*) a plan view showing incident wave heading with respect to the internal cylinder structure. (Online version in colour.)

The internal structure of the cylinder comprised thin vertical barriers which extend to the boundaries of the cylinder and are separated from one another by small uniform gaps through which the fluid is allowed to flow (figure 1). Without loss of generality, the plates are aligned with the x-axis since we allow a plane wave to be incident from infinity at an arbitrary angle, β. The incident wave is of angular frequency ω and wavenumber k and described, under classical water wave theory, by the velocity potential

2.1 $$\phi (r,\theta ,z)=e^{ikr\cos(\theta -\beta )}\psi_{0}(z),$$

(a time-harmonic dependence $e^{-i\omega \tau }$, in which τ is time, is suppressed hereafter) where

2.2 $$\psi_{0}(z)=N_{0}^{-1/2}\cosh k(z+h).$$

In the above, k is related to ω and the depth, h, by the usual dispersion relation

2.3 $$\frac{\omega^{2}}{g}=k\tanh kh.$$

In (2.2) $N_{0}$ is a normalization factor defined by

2.4 $$N_{0}=\frac{1}{2}\left(1+\frac{\sinh2kh}{2kh}\right).$$

Thus $\phi_{\mathrm{inc}}$ is a solution of the governing equation in the fluid,

2.5 $$\nabla^{2}\phi =0,\;-h<z<0,$$

($\nabla^{2}$ being the three-dimensional Laplacian) satisfying the bottom boundary condition,

2.6 $$\phi_{z}=0,\;\text{on }z=-h,$$

and the combined linearized dynamic and kinematic boundary condition,

2.7 $$\phi_{z}-K\phi =0,\;\text{on }z=0,$$

in which $K=\omega^{2}/g$.

Within the cylinder, r<a, −h<z<−d, 0<θ≤2π, additional zero normal flow conditions are to be applied on both sides of each vertical plate within the array. The contrast in lengthscales implied by the assumed small spacing between neighbouring plates relative to both the wavelength and the dimensions of the cylinder allow us to replace the microstructure by the effective field equation (formally derived in Porter [6])

2.8 $${\tilde{\phi }}_{xx}+{\tilde{\phi }}_{zz}=0,$$

where we have written $\tilde{\phi }(x,y,z)\equiv \phi (r,\theta ,z)$. The zero normal flow conditions on the individual elements of the array are taken account of in the derivation of this reduced Laplace’s equation. It is easy to interpret (2.8) as acting to restrict the fluid motion within the gaps in the cylinder to the x−z plane only.

We must also consider how the field within the effective medium governing by (2.8) connects to the fluid outside the cylinder. This can be done formally, but amounts (at leading order in the small parameter on which the derivation of (2.8) is based) to matching the local pressures and local fluxes across the boundary of the cylinder. Thus, on z=−d, r<a, 0<θ≤2π we have

2.9 $$\tilde{\phi }(r\cos\theta ,r\sin\theta ,-d)=\phi (r,\theta ,-d)\;\mathrm{and}\;{\tilde{\phi }}_{z}(r\cos\theta ,r\sin\theta ,-d)=\phi_{z}(r,\theta ,-d).$$

Over the curved surface of the cylinder, r=a, −h<z<−d, 0<θ≤2π, the matching conditions are

2.10 $$\tilde{\phi }(a\cos\theta ,a\sin\theta ,z)=\phi (a,\theta ,z)\;\mathrm{and}\;\cos\theta {\tilde{\phi }}_{x}(a\cos\theta ,a\sin\theta ,z)=\phi_{r}(a,\theta ,z).$$

The term cos⁡θ is geometric and arises from the conservation of mass flux across local triangular matching regions between the channel aligned with the x-axis and the radial flow into r>a. (e.g. [7]).

The only other constraint on ϕ is that it satisfies a standard radiation condition to ensure everything apart from the incident wave is radiating energy towards infinity.

## 3. Solution

The solution of the problem will be expressed using separation of variables inside and outside the cylindrical surface r=a and subsequently completed by matching across r=a appropriately. In r>a, we follow the standard method of expanding the incident wave into polar coordinates using the Jacobi–Anger expansion,

3.1 $$\phi_{\mathrm{inc}}=\sum_{n=-\infty }^{\infty }i^{n}J_{n}(kr)\;e^{in(\theta -\beta )}\psi_{0}(z),$$

in terms of Bessel functions, $J_{n}$, and then the total potential, incorporating waves outgoing to infinity, is written in its most general form as

3.2 $$\phi (r,\theta ,z)=\phi_{\mathrm{inc}}+\sum_{n=-\infty }^{\infty }i^{n}e^{in\theta }\left[a_{n,0}H_{n}(kr)\psi_{0}(z)+\sum_{m=1}^{\infty }a_{n,m}K_{n}(k_{m}r)\psi_{m}(z)\right],$$

where $a_{n,m}$ are Fourier-Bessel expansion coefficients, to be determined, $H_{n}\equiv H_{n}^{\left(1\right)}$ are first-kind Hankel functions and $K_{n}$ are second-kind modified Bessel functions. We have defined depth eigenfunctions by

3.3 $$\psi_{m}(z)=N_{m}^{-1/2}\cos k_{m}(z+h)\;\mathrm{and}\;N_{m}=\frac{1}{2}\left(1+\frac{\sin2k_{m}h}{2k_{m}h}\right),$$

where $k=ik_{m}$ are roots of (2.3) for m=1,2,…, lying on the positive imaginary k-axis: that is, defined by the positive real roots of $K=-k_{m}\tan k_{m}h$ such that $(m-\frac{1}{2})\pi <k_{m}h<m\pi$ for m=1,2,…. In doing so the orthogonality condition

3.4 $$\frac{1}{h}\int_{-h}^{0}\psi_{n}(z)\psi_{m}(z)\;dz=\delta_{n,m}$$

holds for n,m=0,1,2,…, where $\delta_{n,m}$ denotes the Kronecker delta, which is 1 if n=m, and 0 otherwise. The notation has been extended to include (2.2) via $k_{0}\equiv -ik$.

By contrast, determining the solution in r<a is more complicated on account of there being two distinct domains above and within the structured cylinder within which the governing equations differ, their solutions connected by the conditions (2.9).

We write the solution in r<a satisfying (2.5) in −d<z<0 and (2.8) in −h<z<−d in its most general form, being a superposition over all possible wavenumbers and wave angles, thus

3.5 $$\phi (r,\theta ,z)=\sum_{q=0}^{\infty }\int_{-\pi }^{\pi }B_{q}(t)e^{i\mu_{q}(t)r\cos(\theta -t)}Z_{q}(z,t)\;dt,$$

where $B_{q}(t)$ are undetermined functions. The depth variation is defined in a piecewise fashion designed to satisfy (2.6), (2.7) and the matching conditions (2.9) on z=−d, by

3.6 $$Z_{q}(z,t)=\left\{ \begin{array}{ll} \cosh[\mu_{q}(t)z]+(K/\mu_{q}(t))\sinh[\mu_{q}(t)z], & -d<z<0\\ A_{q}(t)\cosh[\mu_{q}(t)(z+h)\cos t], & -h<z<-d \end{array}\right.$$

where

3.7 $$A_{q}(t)=\frac{\cosh\mu_{q}(t)d-(K/\mu_{q}(t))\sinh\mu_{q}(t)d}{\cosh[\mu_{q}(t)(h-d)\cos t]},$$

such that $\mu =\mu_{q}(t)$ are solutions of

3.8 $$\cos t\tanh[\mu (h-d)\cos t]=\frac{K-\mu \tanh\mu d}{\mu -K\tanh\mu d}.$$

For every value of t in [−π,π) there exist an infinite number of discrete roots of (3.8) labelled $\mu =\mu_{q}(t)$ where q=0,1,2,…. It is shown in appendix A that these consist of two real roots labelled $\mu =\pm \mu_{0}(t)$ and an infinite sequence of roots lying on the imaginary axis at $\mu =\pm \mu_{q}(t)$, q=1,2,…. It is also shown in appendix A that there are no roots lying off the real and imaginary axes. We need only include the single positive real root and the sequence of roots that lie on the positive imaginary axis in (3.5) since these contribute to propagating and evanescent waves heading in the direction t, a variable which is integrated over all angles, −π≤t<π.

Using the governing equations satisfied by $Z_{q}$ in −d<z<0 and −h<z<−d, it can be readily shown that these functions satisfy the generalized orthogonality condition

3.9 $$\int_{-d}^{0}Z_{q}(z,t)Z_{m}(z,t)\;dz+\int_{-h}^{-d}Z_{q}(z,t)Z_{m}(z,t)\cos^{2}t\;dz=C_{q}^{2}(t)h\delta_{q,m},$$

on account of $Z_{q}$ being real; $C_{q}(t)$ can be determined explicitly. The identity (3.9) is not used beyond this point, but could be useful in other problems.

It is helpful to employ the Jacobi–Anger expansion of the plane wave function

3.10 $$e^{i\mu_{q}(t)r\cos(\theta -t)}=\sum_{n=-\infty }^{\infty }i^{n}J_{n}(\mu_{q}(t)r)\;e^{in(\theta -t)},$$

so that we have, in place of (3.5), the general expansion

3.11 $$\phi (r,\theta ,z)=\sum_{q=0}^{\infty }\sum_{n=-\infty }^{\infty }i^{n}e^{in\theta }\int_{-\pi }^{\pi }B_{q}(t)J_{n}(\mu_{q}(t)r)e^{-int}Z_{q}(z,t)\;dt.$$

Now ϕ is continuous across r=a for all −h<z<0, 0<θ≤2π. So from (3.2) and (3.11), matching Fourier modes in θ, multiplying by each of the depth eigenfunctions $\psi_{m}(z)$ and integrating over the depth gives

3.12 $$J_{n}(ka)e^{-in\beta }+a_{n,0}H_{n}(ka)=\sum_{q=0}^{\infty }\int_{-\pi }^{\pi }B_{q}(t)J_{n}(\mu_{q}(t)a)e^{-int}F_{0,q}(t)\;dt$$

and

3.13 $$a_{n,m}K_{n}(k_{m}a)=\sum_{q=0}^{\infty }\int_{-\pi }^{\pi }B_{q}(t)J_{n}(\mu_{q}(t)a)e^{-int}F_{m,q}(t)\;dt,$$

where

3.14 $$F_{m,q}(t)=F_{m,q}^{+}(t)+F_{m,q}^{-}(t),$$

and where

3.15 $$F_{m,q}^{+}(t)=\frac{1}{h}\int_{-d}^{0}\psi_{m}(z)Z_{q}(z,t)\;dz\;\mathrm{and}\;F_{m,q}^{-}(t)=\frac{1}{h}\int_{-h}^{-d}\psi_{m}(z)Z_{q}(z,t)\;dz,$$

which can be determined explicitly (see appendix B).

The matching of fluxes across r=a is more complicated since the condition changes across z=−d. In −d<z<0

3.16 $$\phi_{r}{|}_{r=a^{-}}=\sum_{q=0}^{\infty }\sum_{n=-\infty }^{\infty }i^{n}e^{in\theta }\int_{-\pi }^{\pi }B_{q}(t)\mu_{q}(t)J_{n}^{\prime }(\mu_{q}(t)a)e^{-int}Z_{q}(z,t)\;dt.$$

In −h<z<−d we note that

3.17 $$\frac{\partial }{\partial x}e^{i\mu_{q}(t)r\cos(\theta -t)}=(i\mu_{q}(t)\cos t)\;e^{i\mu_{q}(t)r\cos(\theta -t)},$$

and this allows us to write

3.18 $$\begin{array}{rl} & \cos\theta \phi_{x}{|}_{r=a^{-}}=\frac{1}{2}\sum_{q=0}^{\infty }\sum_{n=-\infty }^{\infty }i^{n+1}(e^{i(n+1)\theta }+e^{i(n-1)\theta })\int_{-\pi }^{\pi }B_{q}(t)\mu_{q}(t)J_{n}(\mu_{q}(t)a)e^{-int}\cos tZ_{q}(z,t)\;dt. \end{array}$$

These two expressions are matched over their respective intervals of depth to $\phi_{r}{|}_{r=a^{+}}$ calculated from (3.2) which results in

3.19 $$\begin{array}{rl} k(J_{n}^{\prime }(ka)e^{-in\beta }+a_{n,0}H_{n}^{\prime }(ka)) & \;=\sum_{q=0}^{\infty }\int_{-\pi }^{\pi }B_{q}(t)\mu_{q}(t)\{J_{n}^{\prime }(\mu_{q}(t)a)F_{0,q}^{+}(t)\\ & \;\;+\frac{1}{2}(J_{n-1}(\mu_{q}(t)a)e^{it}-J_{n+1}(\mu_{q}(t)a)e^{-it})F_{0,q}^{-}(t)\cos t\}e^{-int}\;dt \end{array}$$

and

3.20 $$\begin{array}{rl} k_{m}a_{n,m}K_{n}^{\prime }(k_{m}a) & \;=\sum_{q=0}^{\infty }\int_{-\pi }^{\pi }B_{q}(t)\mu_{q}(t)\{J_{n}^{\prime }(\mu_{q}(t)a)F_{m,q}^{+}(t)\\ & \;\;+\frac{1}{2}(J_{n-1}(\mu_{q}(t)a)e^{it}-J_{n+1}(\mu_{q}(t)a)e^{-it})F_{m,q}^{-}(t)\cos t\}e^{-int}\;dt, \end{array}$$

for m=1,2,….

We can now eliminate $a_{n,0}$ between (3.12) and (3.19), the resulting equation holding for −∞<n<∞, and $a_{n,m}$ from between (3.13) and (3.20), resulting in equations for −∞<n<∞ and for m=1,2,…. Combining these results gives rise to the system of equations

3.21 $$\sum_{q=0}^{\infty }\int_{-\pi }^{\pi }B_{q}(t)M_{q,n,m}(t)\;dt=G_{n,m},\;m=0,1,\ldots ,\;n=-\infty ,\ldots ,\infty ,$$

where

3.22 $$G_{n,m}=\delta_{m,0}e^{-in\beta }\left(\frac{J_{n}(ka)}{H_{n}(ka)}-\frac{J_{n}^{\prime }(ka)}{H_{n}^{\prime }(ka)}\right)=\delta_{m,0}\frac{2ie^{-in\beta }}{\pi kaH_{n}(ka)H_{n}^{\prime }(ka)}$$

(after using Abramowitz & Stegun ([11], §9.1.6)) and

3.23 $$\begin{array}{rl} M_{q,n,0}(t) & \;=e^{-int}\{\left(\frac{J_{n}(\mu_{q}(t)a)}{H_{n}(ka)}-\frac{\mu_{q}(t)}{k}\frac{J_{n}^{\prime }(\mu_{q}(t)a)}{H_{n}^{\prime }(ka)}\right)F_{0,q}^{+}(t)\\ & \;+\left(\frac{J_{n}(\mu_{q}(t)a)}{H_{n}(ka)}-\frac{\mu_{q}(t)\cos t}{2k}\frac{\left(J_{n-1}(\mu_{q}(t)a)e^{it}-J_{n+1}(\mu_{q}(t)a)e^{-it}\right)}{H_{n}^{\prime }(ka)}\right)F_{0,q}^{-}(t)\} \end{array}$$

whilst for m=1,2,…,

3.24 $$\begin{array}{rl} M_{q,n,m}(t) & \;=e^{-int}\{\left(\frac{J_{n}(\mu_{q}(t)a)}{K_{n}(k_{m}a)}-\frac{\mu_{q}(t)}{k_{m}}\frac{J_{n}^{\prime }(\mu_{q}(t)a)}{K_{n}^{\prime }(k_{m}a)}\right)F_{m,q}^{+}(t)\\ & \;+\left(\frac{J_{n}(\mu_{q}(t)a)}{K_{n}(k_{m}a)}-\frac{\mu_{q}(t)\cos t}{2k_{m}}\frac{\left(J_{n-1}(\mu_{q}(t)a)e^{it}-J_{n+1}(\mu_{q}(t)a)e^{-it}\right)}{K_{n}^{\prime }(k_{m}a)}\right)F_{m,q}^{-}(t)\}. \end{array}$$

### (a) Numerical approximation

We have to solve (3.21) for the functions $B_{q}(t)$, −π≤t<π for q=0,1,…. Since $M_{q,n,m}(t+2\pi )=M_{q,n,m}(t)$ is a continuous smooth function we assume we can write

3.25 $$M_{q,n,m}(t)=\frac{1}{2\pi }\sum_{p=-\infty }^{\infty }M_{p,q,n,m}e^{-ipt}e^{-i\mu_{q}(t)a},$$

from which it follows that

3.26 $$M_{p,q,n,m}=\int_{-\pi }^{\pi }M_{q,n,m}(t)e^{ipt}e^{i\mu_{q}(t)a}\;dt.$$

The exponential factor involving the argument $\mu_{q}(t)a$ is introduced to suppress the exponential behaviour of the functions $J_{n}(\mu_{q}(t)a)$ for q≥1 when $\mu_{q}(t)$ is imaginary, essential in avoiding numerical solutions becoming dominated by rounding errors. In particular, the NAG libraries, used in our computations, helpfully include the option of computing exponentially scaled Bessel functions. Using (3.25) in (3.21) gives

3.27 $$\sum_{p=-\infty }^{\infty }\sum_{q=0}^{\infty }b_{p,q}M_{p,q,n,m}=G_{n,m},\;-\infty <n<\infty ,\;m=0,1,2,\ldots$$

where

3.28 $$b_{p,q}=\frac{1}{2\pi }\int_{-\pi }^{\pi }B_{q}(t)e^{-ipt}e^{-i\mu_{q}(t)a}\;dt,$$

which implies

3.29 $$B_{q}(t)=\sum_{p=-\infty }^{\infty }b_{p,q}e^{ipt}e^{i\mu_{q}(t)a}.$$

In the above, the assumed expansion of $M_{q,n,m}(t)$ in (3.25) has implied the expansion of $B_{q}(t)$, although an alternative approach would have been to expand $B_{q}(t)$ in terms of *any* basis whose elements are periodic in t with period 2π and determine a discrete system of equations that result. One final computational issue is that the equations nested within (3.18) can be multiplied by $K_{n}(k_{m}a)$ for m≠0 (and $H_{n}(ka)$ when m=0) and this suppresses a second potential source of exponential behaviour of $O(e^{k_{m}a})$ from the elements $M_{p,q,n,m}$.

### (b) The far field diffraction coefficient

As kr→∞ we have from (3.2) and introducing the large argument asymptotics of the Hankel function, that

3.30 $$\phi (r,\theta ,z)-\phi_{\mathrm{inc}}∼{\left(\frac{2}{\pi kr}\right)}^{1/2}A(\theta )e^{ikr-i\pi /4}\psi_{0}(z),$$

where

3.31 $$A(\theta )=\sum_{n=-\infty }^{\infty }a_{n,0}e^{in\theta }.$$

The scattering cross section, representing the total energy in circular waves diffracted by the cylinder, is defined as

3.32 $$\sigma =\frac{1}{2\pi }\int_{-\pi }^{\pi }|A(\theta ){|}^{2}\;d\theta =-\text{Re}\{A(\beta )\},$$

where Re denotes the real part of a complex number, and the last equality follows by the ‘optical theorem’ (Maruo [12] or see Mei [13], eqn (6.33)). Using (3.31) in (3.32) we have

3.33 $$\sigma =\sum_{n=-\infty }^{\infty }|a_{n,0}{|}^{2}=-\text{Re}\left\{\sum_{n=-\infty }^{\infty }a_{n,0}e^{in\beta }\right\}.$$

This latter relation is particularly useful for assessing the accuracy of solutions as it provides two independent calculations, $\sigma_{1}$ and $\sigma_{2}$, say, of the same quantity.

### (c) Forces

The net force acting on the structured cylinder is sum over all of the vertical barriers of the differential pressure acting over each barrier. It is straightforward to determine the effective medium limit of this discrete description which results in the expression

3.34 $$F_{y}=-i\omega \rho \int_{-h}^{-d}\int_{0}^{2\pi }\int_{0}^{a}\phi_{y}(x,y,z)\;r\;dr\;d\theta \;dz,$$

for the hydrodynamic force in the y-direction (there is no component of the force in the x-direction). Using (3.11) combined with an application of y-derivative in the manner suggested by (3.17) gives us

3.35 $$F_{y}=\omega \rho (h-d)\sum_{q=0}^{\infty }\int_{-\pi }^{\pi }\left\{B_{q}(t)\mu_{q}(t)\sin t{\bar{Z}}_{q}(t)\int_{0}^{2\pi }\int_{0}^{a}re^{i\mu_{q}(t)r\cos(\theta -t)}\;dr\;d\theta \right\}\;dt,$$

where

3.36 $$\begin{array}{rl} {\bar{Z}}_{q}(t) & \;=\frac{1}{h-d}\int_{-h}^{-d}Z_{q}(z,t)\;dz=\frac{\cosh\mu_{q}(t)d-(K/\mu_{q}(t))\sinh\mu_{q}(t)d}{\mu_{q}(t)(h-d)\cos t}\tanh[\mu_{q}(t)(h-d)\cos t]\\ & \;=\frac{K\cosh\mu_{q}(t)d-\mu_{q}(t)\sinh\mu_{q}(t)d}{\mu_{q}^{2}(t)(h-d)\cos^{2}t}, \end{array}$$

after using (3.6)–(3.8); we note that ${\bar{Z}}_{q}(t)$ is bounded as t→π/2. Now (3.35) simplifies to

3.37 $$F_{y}=2\omega \rho \pi a(h-d)\sum_{q=0}^{\infty }\int_{-\pi }^{\pi }\{B_{q}(t)J_{1}(\mu_{q}(t)a){\bar{Z}}_{q}(t)\sin t\}\;dt,$$

after using a standard recurrence relation for derivatives of Bessel functions expressed in the form ${\left(xJ_{1}(x)\right)}^{\prime }=xJ_{0}(x)$. Upon using the expansion (3.29) we have

3.38 $$F_{y}=2\omega \rho \pi a(h-d)\sum_{q=0}^{\infty }\sum_{p=-\infty }^{\infty }b_{p,q}\int_{-\pi }^{\pi }\{e^{ipt}e^{i\mu_{q}(t)a}J_{1}(\mu_{q}(t)a){\bar{Z}}_{q}(t)\sin t\}\;dt.$$

For the purposes of presentation, we plot the dimensionless quantity ${\hat{F}}_{y}=F_{y}/F_{\mathrm{cyl}}$ where $F_{\mathrm{cyl}}$ is the force in the y-direction on a solid circular cylinder of radius a extending through the depth (e.g. [14]) subject to waves incident at an angle β to the positive x-axis and given by

3.39 $$F_{\mathrm{cyl}}=\frac{4i\omega \rho h\sin\beta }{kH_{1}^{\prime }(ka)}\left[\frac{N_{0}^{-1/2}\sinh kh}{kh}\right].$$

## 4. Shallow water theory

The truncated structured cylinder protrudes from the bed and acts as a bathymetric metamaterial which can be considered on the assumption that incident waves are long compared to the fluid depth using the shallow water approximation of Marangos & Porter [4]. The approximation is therefore designed to work under the assumptions kh≪1 and a/h≫1 and, consequently, the field variable $\phi_{0}(r,\theta )\approx \phi (r,\theta ,0)$, proportional to the surface elevation, is written in r>a

4.1 $$\phi_{0}(r,\theta )=\sum_{n=-\infty }^{\infty }i^{n}(J_{n}(ka)e^{-in\beta }+a_{n}H_{n}(ka))\;e^{in\theta },$$

where $k^{2}h=K$ defines the wavenumber as the long wave limit of the dispersion relation (2.3).

Inside r<a the governing shallow water equation for the metamaterial depth is, according to Marangos & Porter [4],

4.2 $$\nabla_{xy}\cdot h_{xy}\nabla_{xy}{\tilde{\phi }}_{0}+K{\tilde{\phi }}_{0}=0,$$

where ${\tilde{\phi }}_{0}(x,y)\equiv \phi_{0}(r,\theta )$, $\nabla_{xy}=(\partial_{x},\partial_{y})$ is the two-dimensional gradient and the $\nabla_{xy}\cdot$ is the Cartesian divergence operator. In (4.2)

4.3 $$h_{xy}=\left(\begin{array}{cc} D & 0\\ 0 & d \end{array}\right),$$

is a Cartesian tensor. The full depth of the fluid in r<a is denoted by D and this need not now be the same as h, the depth in r>a.

While the Cartesian description of the field equation is sufficient to generate a general solution within r<a, the boundary conditions on r=a require us to make a transformation into polar coordinates.

### (a) Transformation of the governing equation into polar coordinates

Converting (4.2) into polar coordinates gives us

4.4 $$\nabla_{r\theta }\cdot h_{r\theta }\nabla_{r\theta }\phi_{0}+K\phi_{0}=0,$$

where $\nabla_{r\theta }\equiv (\partial_{r},r^{-1}\partial_{\theta })$ is the gradient and $\nabla_{r\theta }\cdot \equiv (r^{-1}\partial_{r}r,r^{-1}\partial_{\theta })$ the divergence in polars while

4.5 $$h_{r\theta }=\left(\begin{array}{cc} \cos\theta & \sin\theta \\ -\sin\theta & \cos\theta \end{array}\right)h_{xy}\left(\begin{array}{cc} \cos\theta & -\sin\theta \\ \sin\theta & \cos\theta \end{array}\right)$$

is the transformation of the Cartesian depth tensor into polars. Thus we have

4.6 $$h_{r\theta }=\left(\begin{array}{cc} D\cos^{2}\theta +d\sin^{2}\theta & (d-D)\sin\theta \cos\theta \\ (d-D)\sin\theta \cos\theta & D\sin^{2}\theta +d\cos^{2}\theta \end{array}\right).$$

The matching conditions at r=a are: (i) that ϕ is continuous; and (ii) that the depth-averaged flux is continuous. The latter condition is equivalent to

4.7 $$h{\frac{\partial \phi_{0}}{\partial r}|}_{r=a^{+}}=(D\cos^{2}\theta +d\sin^{2}\theta ){\frac{\partial \phi_{0}}{\partial r}|}_{r=a^{-}}+\frac{\left(d-D\right)}{a}\sin\theta \cos\theta {\frac{\partial \phi_{0}}{\partial \theta }|}_{r=a^{-}},$$

(since the flux vector with components in the radial and angular directions respectively is $h_{r\theta }\nabla_{r\theta }\phi_{0}$ in transformed coordinates).

### (b) Solution

This is the analogue of the method used for the full depth-dependent model without the complication of the depth variation. The general solution of (4.2) in r<a can be written

4.8 $$\phi_{0}(r,\theta )=\int_{-\pi }^{\pi }B(t)e^{i\mu (t)r\cos(\theta -t)}\;dt,$$

as a superposition over plane waves travelling in all directions where, in order that the governing equation (4.2) be satisfied,

4.9 $$\mu (t)=\frac{kh}{{\left(D\cos^{2}t+d\sin^{2}t\right)}^{1/2}}=\frac{kh}{{\left(D+(d-D)\sin^{2}t\right)}^{1/2}}.$$

It can be confirmed that (4.8) satisfies (4.6) also. Expanding the complex exponential as a series over Bessel functions (3.10) gives us

4.10 $$\phi_{0}(r,\theta )=\int_{-\pi }^{\pi }B(t)\sum_{n=-\infty }^{\infty }i^{n}J_{n}(\mu (t)r)e^{in\theta }e^{-int}\;dt.$$

We can apply the matching conditions relatively easily now. The matching of $\phi_{0}(a,\theta )$ for 0≤θ<2π results in

4.11 $$J_{n}(ka)e^{-in\beta }+a_{n}H_{n}(ka)=\int_{-\pi }^{\pi }B(t)J_{n}(\mu (t)a)e^{-int}\;dt.$$

The flux condition derived in polars in (4.7) must be used to generate a second relation between the coefficients $a_{n}$ and $b_{n}$ and is clearly more complicated than the first condition. We find, after some algebra that

4.12 $$\begin{array}{rl} kh(J_{n}^{\prime }(ka)e^{-in\beta }+a_{n}H_{n}^{\prime }(ka)) & \;=\int_{-\pi }^{\pi }B(t)e^{-int}\{\left[D+\frac{1}{2}(d-D)\right]\mu (t)J_{n}^{\prime }(\mu (t)a)\\ & \;\;+\frac{\left(d-D)\mu (t\right)}{4}(J_{n-2}^{\prime }(\mu (t)a)e^{2it}+J_{n+2}^{\prime }(\mu (t)a)e^{-2it})\\ & \;\;-\frac{\left(d-D\right)}{4a}((n-2)J_{n-2}(\mu (t)a)e^{2it}-(n+2)J_{n+2}(\mu (t)a)e^{-2it})\}\;dt. \end{array}$$

Bessel function recurrence relations ($J_{n-1}(x)-J_{n+1}(x)=2J_{n}^{\prime }(x)$, and $J_{n-1}(x)+J_{n+1}(x)=2nJ_{n}(x)/x$) provide simplification to

4.13 $$\begin{array}{rl} & kh(J_{n}^{\prime }(ka)e^{-in\beta }+a_{n}H_{n}^{\prime }(ka))\\ & \;\;=\int_{-\pi }^{\pi }B(t)\mu (t)e^{-int}\{DJ_{n}^{\prime }(\mu (t)a)-\frac{i(d-D)\sin t}{2}(J_{n-1}(\mu (t)a)e^{it}+J_{n+1}(\mu (t)a)e^{-it})\}\;dt. \end{array}$$

We eliminate $a_{n}$ from between (4.12) and (4.13) to get

4.14 $$\int_{-\pi }^{\pi }B(t)M_{n}(t)\;dt=G_{n,0},$$

where $G_{n,0}$ is defined by (3.22) and where

4.15 $$\begin{array}{rl} & M_{n}(t)=e^{-int}\{\frac{J_{n}(\mu (t)a)}{H_{n}(ka)}-\frac{\mu (t)}{khH_{n}^{\prime }(ka)}(DJ_{n}^{\prime }(\mu (t)a)-\frac{i(d-D)\sin t}{2}(J_{n-1}(\mu (t)a)e^{it}+J_{n+1}(\mu (t)a)e^{-it}))\}. \end{array}$$

Since $M_{n}(t)=M_{n}(t+2\pi )$ is a smooth function, we can choose to write

4.16 $$M_{n}(t)=\frac{1}{2\pi }\sum_{p=-\infty }^{\infty }M_{n,p}e^{-ipt},$$

which implies

4.17 $$M_{n,p}=\int_{-\pi }^{\pi }M_{n}(t)e^{ipt}\;dt.$$

Using (4.16) in (4.14) gives

4.18 $$\sum_{p=-\infty }^{\infty }b_{p}M_{n,p}=G_{n,0},\;-\infty <n<\infty ,$$

where

4.19 $$b_{p}=\frac{1}{2\pi }\int_{-\pi }^{\pi }B(t)e^{-ipt}\;dt.$$

This approach follows the one we used for the fully depth-dependent formulation in which we have not sought to approximate B(t) directly. The choice of expanding $M_{n}(t)$ in a Fourier basis implies that the solution of (4.18) encodes the Fourier coefficients of B(t) since it follows from (4.19) that

4.20 $$B(t)=\sum_{p=-\infty }^{\infty }b_{p}{\;e}^{ipt}.$$

The two expressions presented in (3.33) can be used as approximations $\sigma_{1}$ and $\sigma_{2}$ to the scattering cross section, σ, with $a_{n,0}$ replaced by $a_{n}$.

The horizontal force on the truncated structured cylinder under the shallow water assumptions is

4.21 $$F_{0y}=-i\omega \rho (D-d)\int_{0}^{2\pi }\int_{0}^{a}\partial_{y}{\tilde{\phi }}_{0}(x,y)\;r\;dr\;d\theta ,$$

in which the depth integral is trivial and the remaining integrals can be addressed in a similar fashion to in §3.3 from which we find

4.22 $$F_{0y}=2\pi \omega \rho a(D-d)\sum_{n=-\infty }^{\infty }b_{n}\int_{-\pi }^{\pi }e^{int}\sin tJ_{1}(\mu (t)a)\;dt.$$

This should be normalized by the shallow water limit of (3.39) in which the value of the square brackets is set to unity.

## 5. Results

In both the full depth-dependent treatment of the problem and the much simpler shallow water approximation, there are a number of numerical parameters whose influence on the accuracy of computations needs to be considered. Thus, range of values of n and p representing the angular variation are reduced to −N≤n,p≤N and the range of values of m and q, representing the vertical variation (or the number of evanescent modes retained), are reduced to 0≤m,q≤M. Apart from these parameters, integrals require numerical approximation. The integrals defined over −π≤t<π are first arranged as integrals over 0≤t<π/2 and then treated using a non-adaptive Gaussian quadrature which is refined to ensure that results are free from quadrature errors up to the sixth decimal place.

It has been confirmed that in the special cases of β=0,π there is no scattering for any a/h, d/h (in addition to D/h=1 in the case of shallow water) and this trivial result is insensitive to truncation parameters N, M. There is no value in presenting numerical results relating to such cases.

Table 1 presents tabulated results that are designed to illustrate the convergence of the numerical scheme with N and M in a non-trivial case. The results are typical of those found for other values of parameters a/h, d/h and β provided either d/h is not too small or a/h is not excessively large. The task is made difficult by the sensitivity in computational stability to increasing values of N and M and neither can become too large without the accuracy of the computations becoming compromised by numerical rounding errors. In particular, increasing N beyond the value of 12 can easily result in numerical errors. Identifying the source of these numerical errors has been difficult. The numerical solution requires the computation of Bessel functions (NAG libraries) of both large argument and large order, the numerical integration of functions which oscillate with increasing frequency with N and have increasingly abrupt changes in $\mu_{q}(t)$ as q increases and the inversion of the complex matrix of increasingly large dimension (2N+1)×(M+1). On the other hand, we can see from table 1 that numerical results converge sufficiently well for presented curves and surfaces to be graphically accurate (i.e. two or three decimal place accuracy) with relatively small values of N and M. The graphical results produced in the paper have used values of N and M within the range of values presented in table 1. Generally, for larger values of ka we require larger N to represent higher frequency diffracted wave effects but generally smaller M. Conversely, when ka is small, N can take small values but M should be larger as the structure influences the fluid motion at larger depths when subject to longer waves.

**Table 1.** Comparison of the two independent calculations of scattering cross section, σ, given in (3.33), against truncation parameters N and M, dictating the number of angular and depth modes, respectively, for two values of kh in the case of a/h=1, d/h=0.5, $\beta =45^{\circ }$.

		kh=1		kh=4	
N	M	$\sigma_{1}$	$\sigma_{2}$	$\sigma_{1}$	$\sigma_{2}$
4	8	0.029682	0.029680	0.006948	0.006924
4	16	0.029678	0.029678	0.006961	0.006932
4	32	0.029694	0.029693	0.006963	0.006935
8	8	0.029724	0.029722	0.006486	0.006471
8	16	0.029645	0.029651	0.006427	0.006441
8	32	0.030404	0.030352	0.006625	0.006861
12	8	0.029704	0.029704	0.006476	0.006469
12	16	0.029662	0.029662	0.006462	0.006456

**Figure 2.**
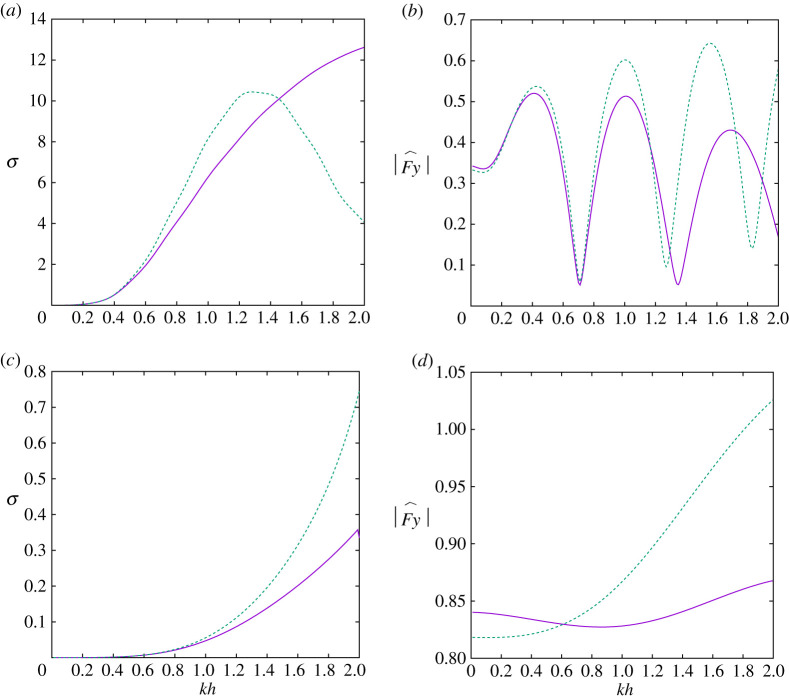
Variation of (*a*,*c*) scattering cross section and (*b*,*d*) dimensionless force with wavenumber kh for $\beta =90^{\circ }$, showing full depth-dependent theory (solid, purple) and shallow water theory (dashed, green). In (*a*,*b*) a/d=4, d/h=0.5 and in (*c*,*d*) a/d=0.5, d/h=0.1. (Online version in colour.)

Figure 2 shows the variation of σ, the scattering cross section, and $|{\hat{F}}_{y}|$, the magnitude of the dimensionless force, computed using full depth-dependent theory and shallow water theory. In the upper two subplots a/h=4, d/h=0.5 and the height of the cylinder one-sixteenth its diameter and in the lower two subplots a/h=0.5, d/h=0.1 and the height is 90% of the cylinder diameter. We see that the shallow water approximation is in good agreement with the full linear theory for smaller values of kh as expected. The shallow water approximation is not designed to work with large and abrupt changes in depth and will therefore tend to work better for short wide cylinders a/h≫1 rather than tall narrow cylinders. The oscillatory behaviour of the force as a function of kh in figure 2*b* is attributed to multiple interference effects of the waves over the top of the cylinder. We note that the force on the structure is of the same order of magnitude as that on an equivalent solid cylinder of the same size.

**Figure 3.**
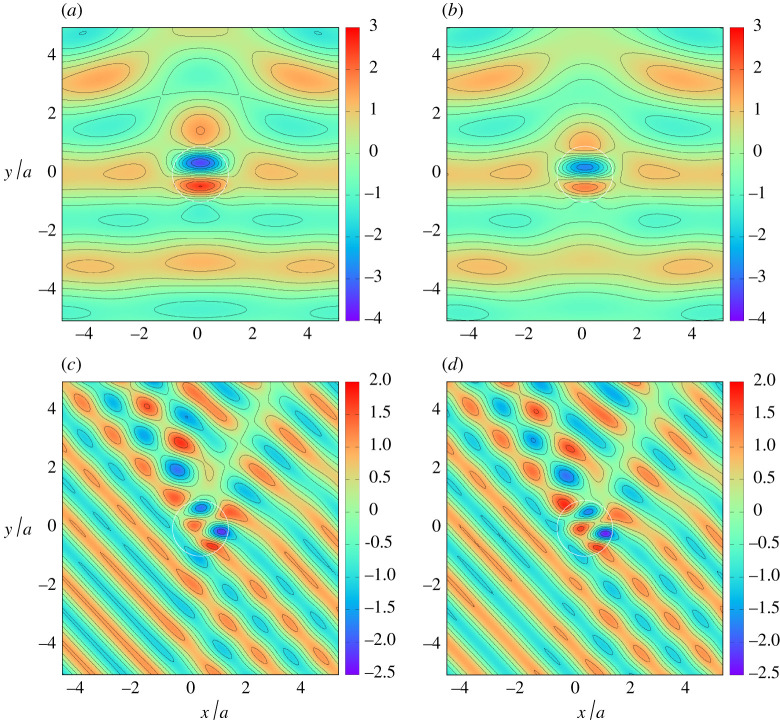
The instantaneous free surface computed using full depth-dependent theory (*a*,*c*) and shallow water theory (*b*,*d*) for a/h=2, d/h=0.1 $\beta =90^{\circ }$, kh=1 in (*a*,*b*) and a/h=4, d/h=0.2, $\beta =45^{\circ }$ kh=1 in (*c*,*d*). (Online version in colour.)

Further comparisons are made between full depth-dependent theory and shallow water theory in figure 3 for kh=1, beyond the wavenumber regime, kh≪1, where shallow water approximation is designed to work. Nevertheless, there is a good qualitative agreement although there are observable differences in the wave elevation particularly in the large amplitudes across the top of the cylinder in the case of d/h=0.1. These large amplitudes are not simply a shoaling effect due to the reduced depth of water; in both plots, we see the signature of the resonance which exists when plates extend fully throughout the depth, as reported by Zheng *et al.* [7]. This near-resonant behaviour is amplified as d/h decreases and is therefore more prominent in figure 3*a*.

**Figure 4.**
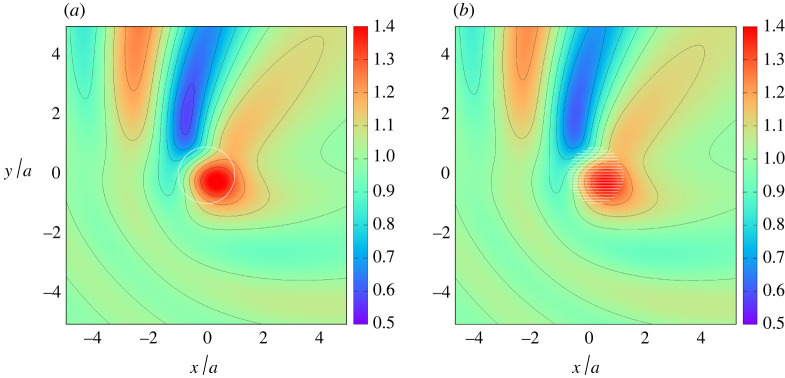
The maximum free surface amplitude computed using full depth-dependent theory (*a*) and using a boundary element method for a discrete plate array (*b*) for a/h=1, d/h=0.2, $\beta =45^{\circ }$ and kh=1.3. (Online version in colour.)

The accuracy of the effective medium model developed in this paper is tested in figure 4 in a comparison with a computation using the boundary element method of Liang *et al.* [15] for an arrangement of discrete thin vertical plates. Boundary element method computations were performed at different wavenumbers and for arrangements of 10, 20 and 40 plates and showed convergence to the effective medium model with increasing numbers of plates. Just one example has been used here for illustration in figure 4 for 20 plates and for a cylinder extending through 80% of the depth. It can be seen that there is visibly almost perfect agreement between the surface plots both inside and away from the cylinder, with the effective medium results predicting slightly more amplification than the discrete computation.

**Figure 5.**
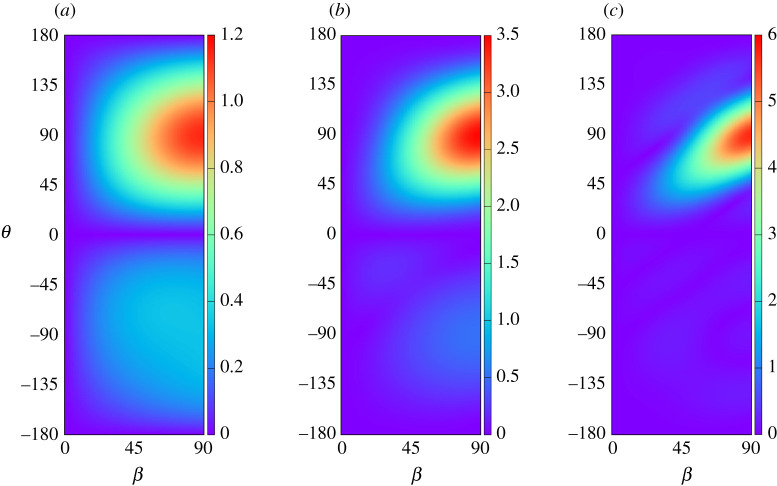
The magnitude of the diffraction coefficient |A(θ)| as a function of diffracted wave angle, θ, and the incident wave angle β for a/h=1, d/h=0.2 and: (*a*) kh=1; (*b*) kh=2; (*c*) kh=4. (Online version in colour.)

The lensing effect of the plate array cylinder is highlighted by the plots in figure 5 showing the magnitude of the diffraction coefficient |A(θ)|, which measures the wave amplitude of circular waves propagating outwards in the direction θ against the incident wave direction β. In figure 5, we adopt the geometry a/h=1, d/h=0.2 used in figure 4 at three wavenumbers, kh=1,2,4. Geometric symmetry implies that only incident wave angles of $\beta \in [0^{\circ },90^{\circ }]$ need be considered. The figures confirm that A=0 when β=0 and also that A(0)=A(π)=0 for all β, which is a consequence of a reciprocity relation (see, e.g. Mei ([13], eqn. (6.34))). As kh decreases below the lowest value of kh=1 shown there is an overall reduction in wave scattering but with increased back-scattering into $\theta \in (-180^{\circ },0^{\circ })$, the diffracted becoming increasingly symmetric about θ=0. The diffraction in the long wavelength limit tends to a dipole and this is confirmed by computations which show that the size of the coefficients, $a_{n,0}$, contributing to outgoing wave propagation, are increasingly dominated by $a_{\pm 1,0}$ as kh→0.

Returning to figure 5 for kh≈1−2 see diffracted amplitudes distributed mainly about a maximum close to $\theta =90^{\circ }$ across a wide range of values of β with decreasing amounts of back-scatter into $\theta \in (-180^{\circ },0^{\circ })$ as kh increases. As kh increases there is a tendency for incident waves to be scattered forwards in the same direction as the incident wave. In all cases, as β approaches $90^{\circ }$ we find the forward scattering at $\theta =90^{\circ }$ reaches a maximum and the magnitude of this scattering increases with kh. The features described above are largely in common with the plate array cylinder extending through the depth described by Zheng *et al.* [7]. Thus, we have shown that the submerged truncated plate array cylinder can be used as a local resonator with the capacity to accumulate wave energy which is transmitted away from the cylinder in a direction perpendicular to the plate array.

**Figure 6.**
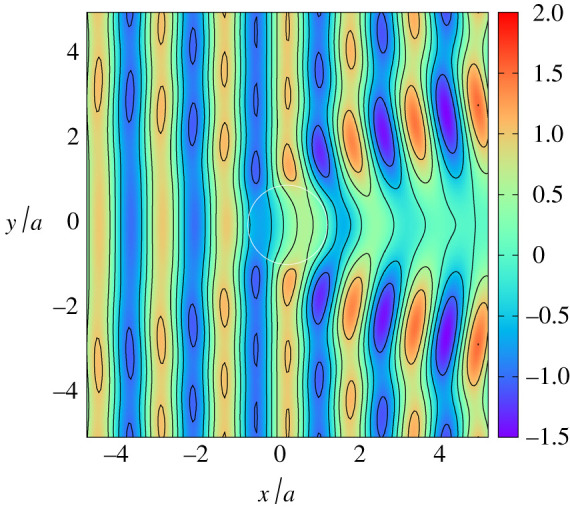
The instantaneous free surface elevation computed under shallow water theory for a/h=4, d/h=1 and D/h=2 with $\beta =0^{\circ }$ and kh=1. (Online version in colour.)

Finally, in figure 6 we showcase the ability of the the shallow water approximation to deal with the case of a cylindrical hollow which extends in r<a to a level D=2h below the depth h in r>a. The hollow is filled with a plate array up to the level d=h. When $\beta =90^{\circ }$ waves of any frequency are transparent to this plate array ‘pit’. In figure 6 where $\beta =0^{\circ }$ there is scattering creating a quiet zone in the lee of the ‘pit’, which is more a function of the lower depth in the pit than due to the presence of the plate array within the pit.

## 6. Conclusion

In this paper, we have considered wave scattering by a porous vertical bottom-mounted cylinder extending part of the way through the depth. The internal structure of the cylinder comprised closely spaced thin vertical barriers whose effect is to confine the motion of the fluid flow in the narrow gaps between the barriers. This allows us to consider that the field inside the truncated cylinder is governed by an effective medium equation with effective matching conditions holding on its boundary. The main focus of the paper has been to develop a solution to the full depth-dependent potential flow problem which exploits this effective medium description of the cylinder. Results have been tested against the shallow water approximation of Marangos & Porter [4] and good agreement is reached for sufficiently long wavelengths as expected. Results are also compared to a boundary element computation of an exact geometrical description involving a finite number of discrete barriers based on the work of Liang *et al.* [15], again showing excellent agreement. Near-resonant wave trapping is promoted within and above the truncated cylinders for sufficiently large wavenumbers. The truncation is important since it implies our solutions do not suffer from undamped resonances reported by Zheng *et al.* [7] for plate array cylinders extending fully throughout the depth. Like the work of Zheng *et al.* [7], we have shown that a by-product of this near-resonant trapping is directional scattering, or lensing, of wave energy perpendicular to the alignment of the plate arrays. New results show that there is no scattering of wave energy in directions parallel to the plate array and that, for long wavelengths (compared to the cylinder radius), the dominant scattering pattern is dipolar. This latter result, which contrasts with the monopolar scattering for small solid cylinders, highlights that small plate array cylinders could be used as a physically realizable element for dipolar scattering in, for example, metamaterial design.

The topic of the current paper and the development of the mathematical methods herein has been considered with a particular, more practical, extension in mind. Work now being considered by the authors involves developing a cylindrical wave energy converter involving a truncated plate array cylinder which intersects the free surface.

## Figures and Tables

**Figure 7.**
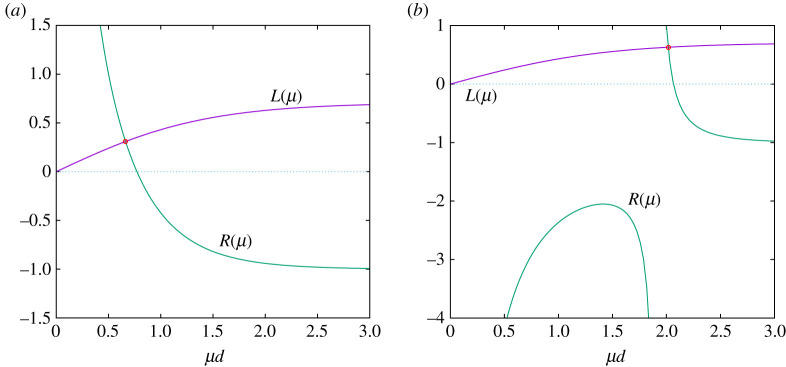
A graphical illustration of location of real roots (circles) for representative examples of h/d=2, t=π/4 and (*a*) Kd=0.5, (*b*) Kd=2. (Online version in colour.)

**Figure 8.**
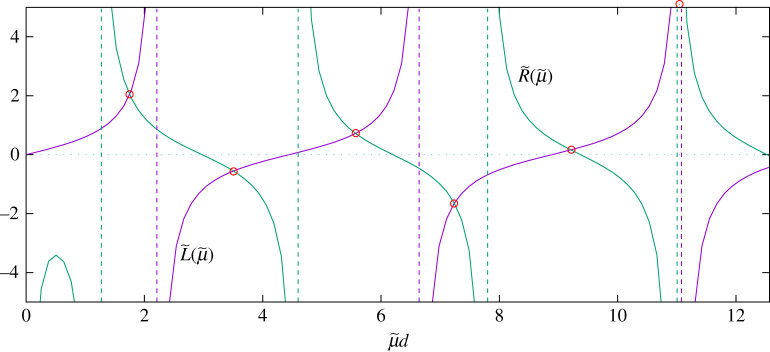
A graphical illustration of location of imaginary roots (circles) for representative examples of h/d=2, t=π/4 and Kd=0.5. The vertical dashed lines represent the asymptotes of the functions $\tilde{L}$ and $\tilde{R}$. There is one root between consecutive pairs of asymptotes. When Kd≥1 the first asymptote of $\tilde{R}$ is at $\tilde{\mu }=0$. (Online version in colour.)

**Figure 9.**
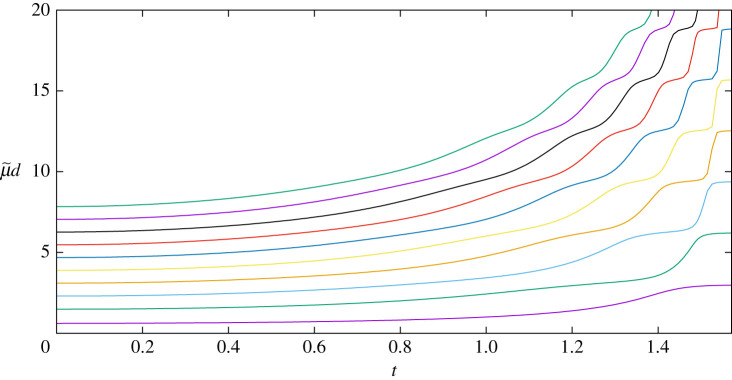
The variation of the modulus of the first 10 imaginary roots, $\tilde{\mu }d$, as a function of t∈[0,π/2] for h/d=4, Kd=0.5. At t=0, t=π/2 the roots coincide with the roots of dispersion relation for a fluid depth h, d (respectively). (Online version in colour.)

## Data accessibility

Numerical code and data files for the results in this article are available to download from https://people.maths.bris.ac.uk/~marp/abstracts/metatrunccyl.html.

## Appendix A. Roots of the dispersion relation

This appendix concerns the roots of (3.8) for −π≤t<π. First, we note that the roots are symmetric about t=0 and t=π/2 and therefore need only be found for 0≤t≤π/2. For t=π/2 the roots of (3.8) coincide with those of K=μtanh⁡μd, while for t=0 the roots coincide with those of K=μtanh⁡μh, the dispersion relation for waves propagating in fluid of depth d and h, respectively. Thus, in these two cases, the locations of roots are well understood (e.g. [16]): there are two real roots and an infinite sequence of imaginary roots, symmetric about real and imaginary axes in the complex plane; there are no complex roots.

The remainder of this appendix concerns the roots of (3.8) for values of t∈(0,π/2) away from these special cases. This is important in developing numerical schemes to ensure all possible roots are captured and can be computed efficiently. We approach this with care since, for example, the dispersion relation corresponding to waves in the presence of a thin floating elastic plate on water of finite depth gives rise to roots which, under certain conditions, move away from the imaginary axes into the complex plane as physical parameters vary. In the elastic plate case, this behaviour is associated with the coalescing of pairs of roots on the imaginary axis [17]. However, in the analysis below we will show that the roots of (3.8) are located on either the real or imaginary axes for all t∈(0,π/2).

This result provides us with our first (and simplest) numerical method for determining roots. Since we are required to compute integrals over 0≤t<π/2 we determine the roots of the water wave dispersion relation for t=0 and then track the roots as t is varied across the integration range, ensuring at t=π/2 the expected roots are accounted for and none have been ‘lost’.

**(a) Real roots**

Consider first that μ is real in

A 1 L(μ)=R(μ),

with

A 2 $$L(\mu )=\cos t\tanh[\mu (h-d)\cos t]\;\mathrm{and}\;R(\mu )=\frac{K-\mu \tanh\mu d}{\mu -K\tanh\mu d}.$$

Both L and R are odd functions, L(0)=0 and L(μ)→cos⁡t as μ→∞ and L is monotonic increasing. Next, R(μ)→K/(μ(1−Kd)) as μ→0 and R(μ)=−1 as μ→∞. If Kd≤1 then the denominator of R, μ−Ktanh⁡μd, vanishes only at μ=0 and R(μ) is monotonic decreasing for μ>0 implying there is one positive root $\mu =\mu_{0}(t)$ (and by extension, one at $\mu =-\mu_{0}(t)$). If Kd>1 then there is a singularity in R(μ) at $\mu =\mu^{*}$ for some $\mu^{*}>0$. But it is easy to show that the only zero of R is at a value of $\mu >\mu^{*}$ and since R(μ)→−∞ as $\mu \to 0^{+}$ in this case, it follows that R(μ)<0 for $0<\mu <\mu^{*}$ and is monotonically decreasing for $\mu >\mu^{*}$. This implies, as before, that there is just one positive real root at $\mu =\mu_{0}(t)>\mu^{*}$, with a symmetric root at $-\mu_{0}(t)$. Both cases are illustrated in figure 7.

**(b) Imaginary roots**

To consider roots of (3.8) lying on the imaginary μ axis let $\mu =i\tilde{\mu }$ where $\tilde{\mu }$ is real such that (3.8) which can be written

A 3 $$\tilde{L}(\tilde{\mu })=\tilde{R}(\tilde{\mu }),$$

with

A 4 $$\tilde{L}(\tilde{\mu })=\cos t\tan[\tilde{\mu }(h-d)\cos t],\;\tilde{R}(\tilde{\mu })=\frac{\tilde{\mu }\sin\tilde{\mu }d+K\cos\tilde{\mu }d}{K\sin\tilde{\mu }d-\tilde{\mu }\cos\tilde{\mu }d}.$$

We note that $\tilde{L}$ and $\tilde{R}$ are odd functions and so if $\tilde{\mu }>0$ is a root then so is $-\tilde{\mu }$.

The function $\tilde{L}$ is an increasing function between asymptotes located at $\tilde{\mu }d=(n-\frac{1}{2})\pi /[(h/d-1)\cos t]$, n=1,2,… and $\tilde{L}$ has zeros at $\tilde{\mu }d=n\pi /[(h/d-1)\cos t]$, n=1,2,….

The function $\tilde{R}(\tilde{\mu })∼K/(\tilde{\mu }(Kd-1))$ as $\tilde{\mu }\to 0$ and either tends to plus infinity (if Kd>1) or to minus infinity (if Kd<1). $\tilde{R}$ has zeros at roots of $\tilde{\mu }\tan\tilde{\mu }d=-K$ (coinciding with the water wave dispersion relation for fluid of depth d) and positive values of these occur once in $(n-\frac{1}{2})\pi <\tilde{\mu }d<n\pi$ for n=1,2,… along the real $\tilde{\mu }$-axis. Finally, $\tilde{R}$ has asymptotes whenever $\tan\tilde{\mu }d=\tilde{\mu }/K$ and these occur on the positive $\tilde{\mu }$ axis for $n\pi <\tilde{\mu }d<(n+\frac{1}{2})\pi$ for n=1,2,…. If Kd<1 there is an additional zero of the denominator at $\tilde{\mu }={\tilde{\mu }}^{*}$ for $0<{\tilde{\mu }}^{*}d<\pi /2$. However, since $\tilde{R}(\tilde{\mu })\to -\infty$ as $\tilde{\mu }\to 0$ in this case and there are no zeros of $\tilde{R}(\tilde{\mu })$ in $0<\tilde{\mu }d<\pi /2$ it follows that $\tilde{R}<0$ for $0<\tilde{\mu }<{\tilde{\mu }}^{*}$. Apart from this exception, $\tilde{R}$ decreases monotonically from plus infinity to minus infinity continuously through zero as $\tilde{\mu }$ increases from one asymptote to the next.

These features are illustrated in figure 8 whence it can be concluded that there are roots of (3.8) between each asymptote of $\tilde{L}$ and $\tilde{R}$ counting $\tilde{\mu }=0$ as the first asymptote when Kd≥1, or the first asymptote of either $\tilde{R}$ or $\tilde{L}$ when Kd<1. Thus discrete roots exist within intervals that are known explicitly and this allows us to solve for roots using, for example, bisection. This provides us with an alternative numerical scheme to the one outlined previously. We have checked that both methods give the same results. An example of how the roots vary as a function of t is provided in figure 9.

**(c) The absence of complex roots**

We consider the possibility that there are values μ∈C satisfying (4.18) which do not lie on either real or imaginary axes. We now write (3.8) in the form

A 5 $$F(\mu )=F_{1}(\mu )+F_{2}(\mu )=0,$$

where

A 6 $$F_{1}(\mu )=R_{1}(\mu )L_{2}(\mu ),\;F_{2}(\mu )=R_{2}(\mu )L_{1}(\mu )$$

and

A 7 $$L_{1}(\mu )=\cos t\sinh[\mu (h-d)\cos t]\;\mathrm{and}\;L_{2}(\mu )=\cosh[\mu (h-d)\cos t],$$

with

A 8 $$R_{1}(\mu )=\mu \sinh(\mu d)-K\cosh\mu d\;\mathrm{and}\;R_{2}(\mu )=\mu \cosh(\mu d)-K\sinh\mu d.$$

Then $F_{1}$ and $F_{2}$ are meromorphic functions. We will use Rouché’s Theorem which states that the number of zeros of $F=F_{1}+F_{2}$ inside a closed contour, C, in the complex plane is equal to the number of zeros of $F_{1}$ inside C provided $|F_{1}|>|F_{2}|$ for all points on C.

We consider the rectangular contour $C=C_{m}(\rho )$, for m=1,2,…, comprised of four straight line segments μ=±imπ/[(h−d)|cos⁡t|]+v, −ρ<v<ρ and μ=±ρ+iu, |u|<mπ/[(h−d)cos⁡t]. Thus ρ and m control the size of the rectangular contour. In order to complete the result, we consider ρ→∞ for each m in Rouché’s Theorem and subsequently allow m→∞.

The choice of the specific distance at which $C_{m}(\rho )$ cuts the imaginary axis is key to the result. Thus it can be confirmed, again most easily by graphical considerations, that between −imπ/[(h−d)cos⁡t] and +imπ/[(h−d)cos⁡t] on the imaginary axis there are as many zeros of $F=F_{1}+F_{2}$ as there are zeros of $F_{1}=R_{1}L_{2}$. For example, referring to figure 8, we choose m=2 in which $C_{m}(\rho )$ cuts the imaginary axis at the second positive zero of $\tilde{R}(\tilde{\mu })$ or $\tilde{\mu }d\approx 8.9$. We can see there are four roots of F, denoted by circles, lying on the positive imaginary axis in the interval $0\leq \tilde{\mu }d≲8.9$. In the same interval, there are two zeros of $\tilde{R}$, coinciding with the zeros of $R_{1}$, and two asymptotes of $\tilde{L}$, which coincide with the zeros of $L_{2}$, and thus four zeros of $F_{1}$. Also, there are exactly two roots of F symmetric placed on the real axis, the same as the number of roots of $F_{1}$ on the real axis ($L_{2}$ has no zeros but $R_{1}$ has two zeros symmetrically placed about the origin; see figure 7).

It can also be shown easily that $|L_{2}|>|L_{1}|$ on $C_{m}(\rho )$ and (less easily analytically, but also confirmed numerically) that $|R_{1}|>|R_{2}|$ on $C_{m}(\rho )$ as ρ→∞ for each m. Thus, $|F_{1}|>|F_{2}|$ on $C_{m}(\rho )$ as ρ→∞ as required by Rouché’s Theorem. Finally, since we know that the zeros of $R_{1}$ only lie on the imaginary axis and the zeros of $L_{2}$ only lie on the real and imaginary axes (being roots of the water wave dispersion equation for water depth d), it must follow that the zeros of F also only lie on the real and imaginary axes.

## Appendix B. Calculation of depth integrals

The explicit expressions for the terms defined by (3.15) are calculated to be

B 1 $$\begin{array}{rl} F_{m,q}^{+}(t) & \;=\frac{-1}{\mu_{q}(t)h\sqrt{N_{m}}(k_{m}^{2}+\mu_{q}^{2}(t))}\{k_{m}\sin k_{m}(h-d)(\mu_{q}(t)\cosh\mu_{q}(t)d-K\sinh\mu_{q}(t)d)\\ & \;\;+\mu_{q}(t)\cos k_{m}(h-d)(K\cosh\mu_{q}(t)d-\mu_{q}(t)\sinh\mu_{q}(t)d)\}, \end{array}$$

with $k_{0}=-ik$ and is simplified by using (2.3) and

B 2 $$\begin{array}{rl} F_{m,q}^{-}(t) & \;=\frac{1}{\mu_{q}(t)h\sqrt{N_{m}}(k_{m}^{2}+\mu_{q}^{2}(t)\cos^{2}t)}\{k_{m}\sin k_{m}(h-d)(\mu_{q}(t)\cosh\mu_{q}(t)d-K\sinh\mu_{q}(t)d)\\ & \;\;+\mu_{q}(t)\cos k_{m}(h-d)(K\cosh\mu_{q}(t)d-\mu_{q}(t)\sinh\mu_{q}(t)d)\} \end{array}$$

again, where $k_{0}=-ik$ and is derived using the dispersion relation (3.8).

Note that, in both expressions, $\mu_{q}(t)$ are pure imaginary when q≥1.

## References

[1] Berraquero CP, Maurel A, Petitjeans P, Pagneux V. 2013 Experimental realization of a water-wave metamaterial shifter. Phys. Rev. E. **88**, 051002. (10.1103/PhysRevE.88.051002) 24329207

[2] Maurel A, Marigo J-J, Cobelli P, Petitjeans P, Pagneux V. 2017 Revisiting the anisotropy of metamaterials for water waves. Phys. Rev. B. **96**, 134310. (10.1103/PhysRevB.96.134310)

[3] Maurel A, Pham K, Marigo J-J. 2019 Scattering of gravity waves by a periodically structured ridge of finite extent. J. Fluid Mech. **871**, 350-365. (10.1017/jfm.2019.259)

[4] Marangos C, Porter R. 2021 Shallow water theory for structured bathymetry. Proc. R. Soc. A **477**, 20210421. (10.1098/rspa.2021.0421)

[5] Zareei A, Alam M-R. 2015 Cloaking in shallow-water waves via nonlinear medium transformation. J. Fluid Mech. **778**, 273-287. (10.1017/jfm.2015.350)

[6] Porter R. 2021 Plate arrays as water wave metamaterials. Wave Motion **100**, 102673. (10.1016/j.wavemoti.2020.102673)

[7] Zheng S, Porter R, Greaves D. 2020 Wave scattering by an array of metamaterial cylinders. J. Fluid Mech. **903**, A50. (10.1017/jfm.2020.660)

[8] Mondal R, Sahoo T. 2012 Wave structure interaction problems for two-layer fluids in three dimensions. Wave Motion **49**, 501-524. (10.1016/j.wavemoti.2012.02.002)

[9] Lawrie JB. 2007 On eigenfunction expansions associated with propagation along ducts with wave-bearing boundaries. IMA J. Appl. Math. **72**, 376-394. (10.1093/imamat/hxm004)

[10] Lawrie JB. 2009 Orthogonality relations for fluid-structural waves in a three-dimensional rectangular duct with flexible walls. Proc. R. Soc. A **465**, 2347-2367. (10.1098/rspa.2009.0066)

[11] Abramowitz M, Stegun IA. 1965 Handbook of mathematical functions. New York, NY: Dover.

[12] Maruo H. 1960 The drift force of a body floating in waves. J. Ship Res. **4**, 1-10.

[13] Mei CC. 1983 The applied dynamics of ocean surface waves. New York, NY: Wiley Interscience.

[14] McCamy RC, Fuchs RA. 1954 Wave forces on a pile: a diffraction theory. *Tech. Memo. No.* **69**. U.S. Army Board, U.S. Army Corp of Eng.

[15] Liang H, Zheng S, Shao Y. 2021 Water wave scattering by impermeable and perforated plates. Phys. Fluids **33**, 077111. (10.1063/5.0051355)

[16] Linton CM, McIver P. 2001 Handbook of mathematical techniques for wave/structure interactions. Southampton, UK: Chapman Hall/CRC Press.

[17] Williams TDC. 2006 Reflections on ice: scattering of flexural gravity waves by irregularities in Arctic and Antarctic ice sheets. PhD thesis, University of Otago, New Zealand.

